# Steroid use in laryngeal diseases

**DOI:** 10.5935/1808-8694.20130025

**Published:** 2015-11-02

**Authors:** Regina Martins

**Affiliations:** Senior Associate Professor of Otorhinolaryngology - Medical School of Botucatu (UNESP); and Coordinator of the Graduate Program in Surgery

In our specialty, steroids are commonly used to treat laryngeal disorders, by different routes of administration; however, in many clinical conditions we still lack scientific evidence enough to prove the benefits of such treatment, as well as the most adequate doses and the best routes for administration. For some laryngeal diseases - such as the supraglottitis, the use of steroids is undisputed - precluding tracheal intubation in adults and children alike. There are some systematic reviews in the literature proving the efficacy of oral dexamethasone and inhaled budesonide or fluticasone propionate in the treatment of supraglottitis^1^, ^2^. Many of these review papers include randomized trials and provide a more reliable assessment based on large samples. Some authors consider the inhalation route as efficient as the oral or intramuscular injection in the treatment of acute inflammatory laryngitis; however, there are those who advocate the oral route of administration as more efficacious. The authors also agree that the side effects of inhaled steroids are less pronounced vis-à -vis those used by other routes.

Another precise clinical indication for steroids is in laryngotracheobronchitis, which involves subglottic edema which often times extends to the tracheobronchial tree. Symptoms of cough, stridor and dyspnea may be minimized with the use of inhaled steroids (budesonide) - considered an efficient and safe treatment.

Steroid indication to prevent post-intubation stridor has also been approached by some systematic review papers and, in such clinical conditions, the intravenous route is the most utilized one. These studies show a trend towards a reduction in the number of re-intubations of newborns, proven reduction in older children, and the lack of evidence that these drugs benefit adult patients^3^, ^4^.

Another very frequent use of steroids is in the course of acute viral laryngitis, especially in people who make professional use of their voices (voice professionals) - singers and actors, who require a quick recovery from their symptoms to return to their professional activities as soon as possible. The steroids used in these cases are usually administered via intramuscular injection (betamethasone) or inhaled^5^.

Despite better patient compliance concerning inhaled treatment, one must stress that it is not free from side effects. Inhaled steroids are commonly used in asthmatic patients, and many of them use the medication day-in day-out for many months. Drug particles, as dry powder, settle along the airway mucosae, and this may cause local irritation, characterizing a form of chemical laryngitis, peaking as inflammation of the airway mucosae. We also notice erythema, mucosal edema, thickening, leukoplasia, granulation tissue and candidiasis^6^, ^7^. The most common symptoms are dysphonia, pain and a burning sensation upon swallowing. These side effects may have negative repercussions on the vocal performance of voice processionals, and symptom improvement is associated with drug-treatment interruption. Thus, these professionals prefer injectable steroids.

Inhaled or intra-lesion steroids have been also used in the remission of some benign lesions of the vocal folds, such as granulomas, nodules and polyps^8^.
